# Mentally Deficient Children

**Published:** 1898-05

**Authors:** Katherine Collins

**Affiliations:** Atlanta, Ga.


### MENTALLY DEFICIENT CHILDREN.

BY KATHERINE COLLINS, M.D.,

ATLANTA, GA.

The subject which I bring before you for consideration is not a new one to you, and my excuse for presenting, is that I believe the mentally deficient child has, hitherto, not engaged the attention of the general practitioner to the extent warranted by the needs of the case; and while it is to the specialist such cases must ultimately be referred, it still remains for the family physician to point the way.

My own personal contact with many children from all grades of society has led me to realize that a closer line should be drawn between the normal and abnormal mind and special attention directed toward the latter, and such disposition made of them as to best promote individual happiness and insure public safety.

For the etiology and classification of these cases, I have drawn largely from the writings of those who have spent years among the defective class and studied it in all its phases.

For the possibilities in the lines of improvement I give you the experience of others, and my own observations.

The earliest efforts to instruct an idiot were made by Itard, of France, in 1800, on a boy found running wild in the woods. Essays, also, upon the subject were written by Voisin and Esquirol, but it remained for Seguin, a pupil of Itard and Esquirol, to make systematic and serious efforts to improve the condition of mentally deficient children.

His first experiments, in 1837, were with the inmates of the Hospital for the Incurables. Later he was appointed to apply his method to the children of the Bicêtre.

Simultaneously with Seguin's work in France, Dr. Saegert, in Berlin, and Guggenbühl, in Switzerland, were making experiments along the same lines, but it was Seguin's great work, entitled "Traitement moral, Hygiène et Education des Idiots et des autres Enfant Arrièrés," that laid the foundation for the emancipation of the imbecile class.

A few years later, in England, a small school for imbeciles was started, others soon followed, while in the United States efforts were being made to benefit the idiots by training them in connection with special schools for the deaf and blind.

Massachusetts was the first State to make specific provision for this class by appointing a commission to investigate the number and condition of idiots in the State. Dr. Howe, instructor of Laura Bridgeman, was chairman, and the report of this commission led to an appropriation of $2,500 for the establishment of a school.

Other States soon followed the example set by Massachusetts, and at the close of 1895 sixteen States made special provision for 6,000 mentally deficient children. The census of 1890 gives a total of 95,571 idiots and imbeciles, leaving 89,571 unprovided for.

England provides for 2,700, and estimates a total of 4,600.

Germany supports 29 institutions, together with the auxiliary schools or "Hülf Klüsse," that are in connection with the public schools proper.

Switzerland, Norway and Denmark also recognize and make provision for these unfortunates.

Such in brief is the historical outline of this work as it has progressed in the countries mentioned.

The term "idiocy" is misleading as it is so variously applied by different writers. Landon Carter Gray defines it as "a congenital condition of mental defect that is technically distinguished from that mental defect of later years which is known as dementia. The symptoms are those of lack of development, and this lack of mental development may vary very much in degree."

The term "idiot" seems in this definition to include all classes of mental deficiency, and to make it always congenital in its origin.

Chas. K. Mills gives three divisions of this class: "Idiots, imbeciles and cretins." He considers idiocy as congenital or acquired in early life previous to the evolution of the mental faculties.

While the division into the above classes is often confusing, the division as to time of acquisition is very definite, separating it entirely from those cases occurring in later life, such as dementia. Mills uses the terms imbecility and feeble-mindedness interchangeably.

Shuttleworth, of England, includes all grades under the term "mentally deficient," and groups them according to time of acquisition with a pathological subdivision. Like all classifications, it is more or less imperfect, but it gives a convenient working outline and is adopted by many institutions.

The first division is that of *congenital origin*, under which head we have the microcephalous, hydrocephalous, mongol, scrofulous birth palsies, cretinism and primarily neurotics.

These forms may exhibit any degree of mental defect from the low grade idiot to the merely backward child, according to the extent to which the primary cause acts.

To enable you to understand the table better I will call your attention to a few points in each case, but for a fuller description will leave you to refer to any good text-book upon the subject.

The microcephalic in its extreme form is characteristic of low grade idiocy. An attempt was made at one time to establish, in these cases, seventeen inches as a limit of circumferential measurement; such a limitation seems not to be wholly satisfactory, though Fletcher Beach states that heads measuring less than this never show any signs of intelligence. The diagnosis, however, of this condition is not based upon measurement alone; the characteristic receding forehead, pointed vertex, flattened occiput, with the bodily peculiarities, will not leave one long in doubt as to which class the subject belongs.

Hydrocephalous is occasionally congenital and does not always produce mental enfeeblement. Distinction must be made between this condition and rickets; in the latter the fontanelle is depressed and the head elongated in the antero-posterior diameter, while in the former the fontanelle is raised and the crossed diameters more nearly approximate.

In the Mongol or Kalmuc type, so named from a strong resemblance to these races, the skull is short, the transverse and longitudinal diameters nearly equal, the frontal and occipital planes are almost parallel; the tongue is transversely fissured and has hypertrophied papillæ; the eyes are frequently set obliquely. Antopsies in these cases show the brain to be made up of coarse convolutions.

Shuttleworth and Beach agree that the Mongol variety is often the last born of a long family with a tubercular family history, and it is not unusual for them to die in early life of tubercular affections.

Cretinism, which is characterized both by the lack of physical and mental development, when it occurs *in utero* results in death to the fetus, but in some cases the congenital taint is developed after birth. These cases are recognized by the low stature, large head in proportion to the body, flat on top, narrow in front and spreading out toward the sides, the hair is coarse and bristle-like, the forehead low, the skin loose and rough.

The thyroid gland is absent and fatty tumors are formed in the supra-clavicular region. The Cretin is usually of a low grade of mentality but not vicious; they have rather an affectionate disposition, can recognize their friends, and seem in some cases to understand a little of what is going on about them.

According to the experiments of Dr. Telford Telford-Smith of England, improvement has followed the administration of the thyroid extract in cases of sporadic cretinism, and reversion to the former condition occurs upon withdrawal of the remedy. Removal from the low valleys, where these cases occur, to higher altitudes seems to have a modifying influence.

Birth palsies will be considered under traumatic injuries to the new-born.

Of the next division little need be said, as it is a familiar condition.

Dr. Ireland claims that two-thirds of the idiots show scrofulous diathesis; strumous glands, tubercular disease of the joints and serous membranes are frequent accompaniment of mental defection.

The next division is the primarily neurotics. I am rather inclined to take issue with some authors upon this point. My experience leads me to believe that while mental enfeeblement may show itself in the child where parents are neurotics, yet there is oftener a condition of instability of the general nervous system accompanied by a high grade of mental development. Should, however, this neurotic state be grafted upon an epileptic, tubercular and other depraved condition, then mental enfeeblement of any degree may result.

The second general class, Developmental, is where a latent or unstable condition of the central nervous system exists, congenital in origin, but not exhibited until precipitated by some crises.

Convulsions during the first or second dentition and the changing conditions of puberty often mark the downward path of an already tottering mind. Epileptic seizures frequently do not begin until the sixth or seventh year, and up to that age the child may seem as bright as other children.

In those cases of inherited syphilis the characteristic lesions may occur early, but mental deterioration rarely begins before the second dentition.

The third general class consists of those cases which are *non-*

*congenital,* and under this head we have traumatic, post-febrile-shock, and toxic cases.

Traumatism may act at the time of birth or later. A severe fall in early infancy, producing injuries resulting in pressure upon the brain or causing meningeal hemorrhage, may result in degeneration of the brain substance.

As to traumatism at the time of birth, much has been said and written. Dr. J. Madison Taylor, of Philadelphia, several years ago carefully collected the opinions of many specialists regarding the number and extent of cerebral injuries due to forceps deliveries. I will give briefly some of the replies he received:

Jacobi writes as follows: "That while forceps may do injury by the fact that the very presence of the blade narrows the canal, but the greater danger arises from the prolonged labors."

Drs. Sarah McNutt of New York, Fullerton of the Woman's Hospital, Philadelphia, Martin of Ann Arbor, Davis, Sinkler, and Hirst of Philadelphia, look upon prolonged labors as the more fruitful source of these cerebral injuries in the new-born.

Dr. Wm. Goodell, however, is quoted as follows: "My experience would lead me to the belief that the great majority of cases of cerebral palsies are due to acute unequally distributed pressure of the forceps upon the child's head, rather than the prolonged but equally distributed pressure in an unaided labor."

Dr. Joseph Price states that during his stay at Preston Retreat he did not have a direct or remote cerebral sequela of parturition in infants, though there were many complicated labors, some with contracted pelvis. Dr. Price fiercely denounces the promiscuous and unskilled use of the forceps.

Post-febrile conditions producing meningeal inflammation may cause more or less deterioration or arrest of development. Shock in the form of fright is sometimes assigned as the origin of mental defect, and where it is prolonged may, by interference with nutrition of the nerve centers, bring about trophic changes more or less permanent in character.

Toxic cases are seen in very young children that are subjected to long-continued use of alcohol or narcotics.

Instead of going into a long discussion of the etiology of mental deterioration, I will run over a few tables that will, I think, give you a better idea of the subject than any words of mine.

The first is

Beach and Shuttleworth.—Royal Albert Asylum, 1200 cases.

I. CAUSES ACTING BEFORE BIRTH.

| | | | |
|---|---|---|---|
| Phthisis | 291 | Intemperance | 159 |
| Mental strain | 191 | Syphilis | 16 |
| Insanity | 182 | Illegitimacy | 23 |

II. CAUSES ACTING AT BIRTH.

| | | | |
|---|---|---|---|
| Primogeniture | 228 | Instrumental delivery | 39 |
| Protracted pressure | 57 | Premature birth | 37 |

III. CAUSES ACTING AFTER BIRTH.

| | | | |
|---|---|---|---|
| Infantile convulsions | 391 | Head injury | 99 |
| Acute infectious fevers | 119 | Epilepsy and cerebral affections | 57 |

Dr. Pearce, of Philadelphia, has, from the statistics of many institutions compiled several very valuable tables. I will only quote the one giving the causes assigned for the mental enfeeblement.

| CONGENITAL. | Pater. | Mater. | ACQUIRED. | |
|---|---|---|---|---|
| Imbecility | 16 | 21 | Accident | 75 |
| Inebriety | 10 | 11 | Abuse and neglect | 10 |
| Nervousness | 17 | — | Infantile disease | 95 |
| Epilepsy | 3 | 30 | Sunstroke | 5 |
| Hysteria | — | 5 | Instrumental delivery | 25 |
| Shock | — | 25 | | |
| Insanity | — | 7 | | |
| Overstraining during gestation | — | 40 | | |
| Syphilis | — | 7 | | |
| Phthisis and consanguinity | — | 17 | | |

The incongruity between the number ascribed to imbecility, alcoholism, or any other cause that would reflect discredit to the parents, and the number ascribed to overstraining, shock, and injury is always very marked in these tables, and allowance must be made accordingly.

A few words as to the moral imbecile before taking up the disposition of the mentally deficient: He belongs to a class apart from the others. Frequently the mental powers are good, but totally lacking in moral sense and not amenable to any teaching in this direction. Punishment and persuasion alike fail and he has no moral sense to appeal to. He is the despair of his parents and teachers, and his influence among other children is bad. Lifelong detention is at present the only disposition that can be made of such cases.

We will now take up the management of the mentally deficient as it is exhibited by the special institution. The late Dr. Kerlin was very active in initiating the work in this country that has proven so beneficial to the public at large as well as the individuals. In olden times the Greeks destroyed their "fools," but in this Christian age we are not permitted to resort to such extreme measures, so it remains for us to protect the people and promote the happiness and usefulness of the individual by educational means.

In the many institutions provided for this class, the school system takes precedence over the custodial. In fact the entire institution might be considered a school, inasmuch as some degree of training is (with few exceptions) resorted to in all cases. The faintest ray of intelligence is carefully looked for, and when found acts as a lever to open the mind to other impressions.

The hand is generally the first to receive attention. The useless hand of the idiot is familiar to all. The simple act of raising a cup of water to the lips is often denied it; but, after training, many of these children can make beds, sweep, and perform other domestic duties. Every possible means is resorted to, to at least raise the independence of these children, who cannot perform the simple acts of daily life that the normal child does continually, unconscious that it ever had to learn to do just that one thing.

Sometimes a child is labored with for years in this simple way until he has reached a point of development where he can enter into the kindergarten work; from there take up the sloyd or manual arts, on into what corresponds to the primary and intermediate grades of our public schools. Here at the age of twenty years you will find him with a fair amount of knowledge of geography, arithmetic, spelling, etc., able to perform in a very intelligent way rough carpentering work, gardening, etc. It is possible now for him to be sent out to earn his own livelihood, providing he be placed under proper supervision and not subjected to temptation. Many of these cases are not primarily vicious, but often show a sweet, lovable nature, capable of much good or evil, according to the influence that surrounds them. The will is weak, and they become an easy tool to their more evil companions.

Institution work is organized upon a broad scale to meet every requirement. Military drill and calisthenics receive special attention, and together with all manual work, prove important factors, for mental specialists generally agree that the education of the body helps to develop the mind.

A band is usually formed from the inmates. Entertainments are given at intervals wherein the children take active part. Sewing and all domestic duties are taught, together with gardening, carpentering and the general care of the premises. As a consequence the number who are absolutely helpless is greatly reduced.

How much better this condition than the retaining of such a child in the home where it degenerates instead of improves, bringing trouble and anxiety to all about him.

Many parents would send such children to special institutions, but are debarred from doing so through financial reasons, and as yet comparatively few States make provision for them. Others are deterred from doing so through a false sentiment; this would be overcome if the institutions were considered more in the light of schools than asylums.

There is a class of the mentally defective found in our schools which represent the borderline cases, recognized oftener by the teacher than the physician. Such cases exhibit incapacity in one or more directions, being normal in other respects and often excel in some one line. These children, while capable of receiving an education, cannot be treated as the normal child. School authorities realizing this, are making efforts to provide special instruction (as is done in Germany) for them in connection with the city schools.

Every city should have an "auxiliary school" centrally located, where these children can receive individual training without being sent away from home. In some instances after a year or two of special training a child may be permitted to take its place in the regular school work.

These children's minds are like some plants; they need more tender care to start them growing, but when once a foundation is laid, become vigorous and hardy; but if deprived of this initial care, grow up weak and distorted.

The anemic child must not be confounded with actual mental defect, for in the former the sluggishness of mental activity is a general condition dependent upon improper nutrition, and such a child should not be in school at all, but given proper food and outdoor life until the anemic condition is overcome.

In closing I would say a few words concerning State economics as related to this subject. The providing for these cases by the establishment of State institutions for the severer cases and auxiliary schools for the milder ones means, of course, a large expenditure of money, but one function of the State is to protect and care for its own, and after all is it not better and more economical to support such institutions for the improvement of a class than penitentiaries and poorhouses for the detention of the criminal and disabled? Sooner or later a large proportion of the mentally deficient become wards of the State in one capacity or another.

